# The Positive Side of Maximization: Linking Maximization Tendency With Meaning in Life Through Time Perspectives

**DOI:** 10.3389/fpsyg.2021.708117

**Published:** 2021-10-04

**Authors:** Min Ma, Na Zhao, Li Zhang

**Affiliations:** Department of Psychology, Institute of Sociology and Psychology, Central University of Finance and Economics, Beijing, China

**Keywords:** maximization, time perspective, meaning in life, past-positive, future

## Abstract

The negative influence of maximization on well-being, that is, the maximization paradox, has received increased attention. However, few studies have shown the link between maximization tendency and meaning in life, which is one type of well-being, and no empirical literature has examined the mechanisms between them. We conducted an online survey in China to test the relationship between maximization tendency and meaning in life. Participants (*N*=2,987) were invited to report their maximization, time perspective, meaning in life, and other control variables and demographic variables. Multi-mediation path analysis was adopted in the data analysis. The results revealed that maximization was positively associated with meaning in life, which confirmed the positive aspect of the maximization tendency. Further analyses indicated that the time perspectives of past-positive and future mediated the positive relationship between maximization and meaning in life. In contrast, a present-fatalistic time perspective was a suppressor in the positive relationship. Our findings suggest that the maximization tendency has a positive aspect rather than the overall maximization paradox. An important means of elevating meaning in life is to encourage the time perspective of past-positive and future-oriented and reduce the present-fatalistic time perspective.

## Introduction

Considerable studies have addressed that decision styles have important influence on individuals’ well-being and feelings at the end of the decision process (Iyengar and Lepper, 2000; Schwartz et al., 2002). Two typical decision styles are differentiated, including maximizing and satisficing during the daily choice process (Simon, 1956). A maximizer tends to seek the best option in a decision situation, spending a great amount of effort, and delving into the alternative search of all possibilities to find the very best option in a decision situation, while a satisficer will stop the decision process and accept goods that are good enough to satisfy the certain individual threshold (Schwartz et al., 2002). A large number of studies have found that individuals with a maximizing decision-making style tend to experience more negative emotions and consequences in the decision-making process (Schwartz et al., 2002; Iyengar et al., 2006; Kim and Miller, 2017; Newman et al., 2018) which is termed as the “Maximization Paradox” (Dar-Nimrod et al., 2009). However, most studies have addressed association between subjective well-being (SWB) and maximization. Empirical studies investigating the relationship between psychological well-being (PWB) such as meaning of life and maximization are sparse. To fill this gap, this study was devoted to examine the relationship between maximization and meaning in life and to identify the mechanism underlying this relationship.

## Literature Review

A body of studies have found that maximizers tend to experience negative emotions (Schwartz et al., 2002; Iyengar et al., 2006; Kim and Miller, 2017) in the decision-making process, that is, the maximization paradox (Dar-Nimrod et al., 2009), either in the general domain or in the specific domains. For example, in a general decision-making domain, maximization was negatively correlated with happiness and life satisfaction across distinct samples (Schwartz et al., 2002). In the domain of the job search process, compared to satisficers, maximizers experienced more negative affect and were less likely to be satisfied with the jobs they obtained (Iyengar et al., 2006). In the domain of friendship selection, Newman et al. (2018) examined the negative link between maximizing and life satisfaction and found that attempts to maximize are harmful to one’s well-being. In short, these studies focus on the relationship between maximization and well-being jointly either in the general domain or in the specific domains.

Studies have examined the mechanisms of this negative relationship. In sum, a series of cognitive biases are helpful to understand this paradox, including a belief in an objective best (Luan and Li, 2017), higher cost (Iyengar et al., 2006), higher expectation (Dar-Nimrod et al., 2009), and loss aversion (Polman, 2010). Additionally, the low ability to handle dissonant experience is a key point. For instance, Kim and Miller (2017) showed that maximizers are unhappier decision-makers than satisficers because maximizers fail to handle dissonant experiences adequately.

However, the negative relationship is not always in case. One study by Datu (2016) examined the relationship among positive emotion, maximization, and meaning in life in a collectivist context with Filipino college students. The findings showed that maximization moderated the link between positive affect and meaning in life, suggesting the positive relationship between maximization and meaning in life.

Meaning in life is a crucial component of human well-being. Literature reviews suggest that there are two kinds of well-being: SWB referring to the emotional and momentary experience of positive affect or happiness and a cognitive judgment on satisfaction with one’s life as a whole or with specific life domains and PWB referring to meaning in life self-realization (Ryan and Deci, 2001; Delle Fave et al., 2011; Henderson and Knight, 2012). The former is conceptualized as the predominant view of hedonism and the latter as eudaimonism (Ryan and Deci, 2001). Researchers generally agree that meaning in life implies a sense of existence, a sense of purpose in one’s life, and a sense of fulfillment (Ho et al., 2010).

Taken together, both life satisfaction and meaning in life are vital indicators of well-being. The neural substrates and biobehavioral correlates of well-welling have been broadly revealed, and the increasing evidence supports that well-being is a protective factor of physical and psychological health (Davidson, 2004; Ryff, 2014). Compared with the plenty studies about the link between maximization and life satisfaction, little research is devoted in the relationship between maximization and meaning in life (Datu, 2016). Meaning in life is a neglected aspect of human well-being. Some researchers have asserted that the sense of meaninglessness in life will cause distress and leave many people vulnerable to mental health problems and possible suicidal behavior (Galek et al., 2015; Glaw et al., 2017). This study aimed to examine the link between maximization tendency and meaning in life.

Although no evidence revealed the direct relationship between maximization and meaning in life, it can be inferred that they have may link indirectly.

First, maximizers pay more attention to the future consequences of their present actions due to high standards and tend to hold the mindset of future-oriented thinking (Zhu et al., 2017). Future-oriented thinking is defined as the current anticipation of future goals and the results of present actions (Howlett et al., 2008). This thinking style implies individuals take a future time perspective. Time perspective (TP) is originally emphasized as a vital antecedent to behavior, emotion, and motivation (Lewin, 1951). It describes stable individual differences in the construction of temporal experience and is “a fundamental dimension in the construction of psychological time, emerges from cognitive process portioning human experience into past, present, and future temporal frames” (Zimbardo and Boyd, 1999, p. 1271). The Zimbardo Time Perspective Inventory (ZTPI) is a theory-based instrument including the motivational, emotional, cognitive, and social processes determining the TP (Zimbardo and Boyd, 1999). It includes five types, that is, past-positive, past-negative, future, present-hedonistic, and present-fatalistic. Past-positive refers to a sentimental and warm attitude toward the past, past-negative reflects an integrated negative view of the past, future perspective refers to a future-oriented attitude, present-hedonistic reflects a hedonistic and risk-taking attitude, and present-fatalistic refers to a fatalistic and hopeless attitude toward the future and life (Zimbardo and Boyd, 1999). Recent research has proposed a balanced time perspective (BTP) which is defined as a tendency to think about past, present, and future in positive ways (Zimbardo and Boniwell, 2004; Webster, 2011). Time in those individuals with BTP is expansive but not restrictive (Webster, 2011). The BTP is theorized as a combination of high scores on past-positive, present-hedonic, future dimensions, and low scores on the past-negative and present-fatalistic dimensions (Zimbardo and Boniwell, 2004). TP as “a cognitive operation that implies both an emotional reaction to imagined time zones (such as future, present or past) and a preference for locating action in some temporal zone” (Lennings, 1996, p. 72), it is likely to be used for time management for maximizers to achieve their goals. For example, with higher standards, maximizers will construe time more distally and therefore be more likely to take a future time perspective (Trope and Liberman, 2010).

Second, comparing a plenty of experimental and neuroscience studies about time perspective and SWB (Farb et al., 2007; Desmyter and De Raedt, 2012; Stolarski et al., 2014, 2016), a few studies have examined the association between time perspective and meaning in life and they have revealed a close association between time perspective and meaning in life (Steger et al., 2008; Leshkovska and Shterjovska, 2014; Webster et al., 2021; Zheng and Wang, 2021). Meaning in life has a strong temporal basis (Webster et al., 2021). Steger (2012) states that “meaning in life researchers presume they are studying a fundamental orientation of the person to the world, embracing all that is important and vital to someone’s past, present, and future” (p. 382). Meaning in life was often found to be positively correlated with past-positive and future but negatively correlated with past-negative (e.g., Steger et al., 2008; Leshkovska and Shterjovska, 2014; Zheng and Wang, 2021). Sobol-Kwapinska (2009) found those with higher present-fatalistic have a lower sense of meaning in life. In the study by Zheng and Wang (2021) with Chinese youngsters as subjects, present-fatalistic was negatively correlated with meaning in life, while no reliable association was observed between meaning in life and present-hedonistic time perspective. In summary, the close association between time perspective and meaning in life and make it possible that maximization on meaning in life may be mediated by time perspective.

## Current Study

There are two objectives in the present study. The key objective of the current research was to examine the relationship between maximization and meaning in life. To our knowledge, little literature has examined the relationship between the maximization and meaning in life (Datu, 2016). In the study by Datu (2016), a positive association between the maximization tendency and meaning in life was observed. Different from the study by Datu (2016), our study investigated the direct relationship between maximization and PWB, that is, meaning in life in the Chinese culture. To enrich literature about the “maximization paradox” and manage the psychological sequelae of decision styles, it is important to identify the valence of the link between maximization tendency and meaning in life. We hypothesized that there is a positive association between the maximization tendency and meaning in life. One study showed that high standard, which is one dimension of maximization tendency is positively correlated with meaning in life (Park and Jeong, 2016).

A second important objective was to investigate the underlying mechanism linking the maximization tendency and meaning in life. We hypothesize maximization tendency is correlated with meaning in life through time perspectives.

First, we hypothesize that past-positive and future, rather than past-negative, tending to enhance the purpose of life, is positively correlated with meaning in life. Meaning in life depends on two key aspects of time perspective, including past and future time perspective (Waytz et al., 2015) according to construal level theory (Trope and Liberman, 2010). This theory stated that higher standards were evaluated more distantly regarding time, and we hypothesized that the association between maximization and meaning in life was mediated by past and future time perspectives. Further, positive emotion is more likely to enhance meaning in life from the perspective of the broaden-and-build theory (Fredrickson, 2013). Thus, we hypothesize that past-positive and future, rather than past-negative, tending to enhance the purpose of life. This hypothesis can be supported by some empirical studies (Hicks et al., 2012; Waytz et al., 2015) which showed that the relatively high level of past-positive and future played pivotal roles in the formation of meaning in life. With a positive past or future time perspective, individuals built new patterns or explanations of the events. These patterns or explanations become an integral part of their lives and improve the probability that these individuals will endorse and construct more meaning with the chosen behavior.

Second, we hypothesize that a present-fatalistic time perspective plays a suppressed mediating role between maximization and meaning in life. In detail, we suppose that a present-fatalistic time perspective is negatively correlated with meaning in life as it tends to aggravate a sense of worthlessness. Studies have indicated that a present-fatalistic time perspective is a key factor in predicting PWB (Díaz et al., 2015). An empirical study explained the possible mechanism of this link from the perspective of perceived control (Díaz et al., 2015). Given that the lack of perceived control is one of the central features of fatalism and perceived control is negatively correlated with meaning in life (Stavrova et al., 2020), it seems reasonable to conclude that fatalism is negatively related to meaning in life. In short, we hypothesize that a present-fatalistic attitude plays a mediating role between maximization and meaning in life; however, the valence is distinct from past-positive and future time perspectives.

To achieve the two intended purposes, we ran a survey in 31 provinces of China. Our first hypothesis is that there is a positive relationship between the maximization tendency and meaning in life. The second hypothesis is that there are three parallel routes between maximization tendency and meaning in life. That is, past-positive and future time perspectives mediate the positive relationship between maximization and meaning in life, while a present-fatalistic perspective suppresses the positive relationship between them. In other words, past-positive and future time perspectives could account for the positive relationship between them. Also, as the data were collected during the COVID-19 crisis and research indicated that maximization was correlated with risk perception and risk tendency (Qiu et al., 2020), we used a few risk perception indicators (exposure risk level, perceived threat and coping efficacy) as control variables.

## Materials and Methods

### Participants and Procedures

The sample comprised 3,459 participants from 31 provinces in China. We excluded 471 participants who had not completed the survey seriously. Data collection took place from 3 to 13 March, 2020. Data were collected *via* self-reporting questionnaires using the online survey platform Sojump.^1^ Informed consent was obtained from all of the participants, and the questionnaires took about 15min to complete. The participants received 10 Yuan for completing the survey. This study has been approved by the School of Sociology and Psychology Academic Committee of Central University of Finance and Economics.

### Measurements

#### Maximizing Scale

The maximization tendency was assessed based on the 13-item Maximization Scale (Schwartz et al., 2002). The scale includes three dimensions, namely, alternative search (e.g., “I often fantasize about living in ways that are quite different from my actual life”), decision difficulty (e.g., “When shopping, I have a hard time finding clothing that I really love”), and high standards (e.g., “I never settle for second best”). The participants answered items on a 7-point Likert scale ranging from 1 (complete non-conformance) to 7 (complete conformance). The consistency of the scale is good, and the consistency coefficients of the three dimensions of alternative search, decision difficulty, and high standards are 0.80, 0.82, and 0.80, respectively. We averaged the ratings to obtain the overall score. A higher score indicated that the individual has a higher maximizing tendency, and a lower score indicated that the individual has a lower maximizing tendency. Common factor analysis (CFA) indicated that the model fits the data well: *χ*^2^(62)=792.30, *χ*^2^/*df*=12.78, comparative fit index (CFI)=0.95, Tucker–Lewis index (TLI)=0.94, root mean square error of approximation (RMSEA)=0.06 (90% CI: 0.059–0.067), and standardized root mean square residual (SRMR)=0.034. The factor loadings of the items range from 0.59 to 0.78. In this study, this measure demonstrated good internal consistency (*α*=0.89).

#### Zimbardo Time Perspective Inventory

Time perspective was assessed based on the Chinese brief version (Lyu et al., in preparation; Wang, 2016) of the ZTPI (Zimbardo and Boyd, 1999), including five dimensions in total, namely, past-negative (e.g., “Painful past experiences keep being replayed in my mind”), present-hedonistic (e.g., “I do things impulsively”), future (e.g., “I complete projects on time by making steady progress”), present-fatalistic (e.g., “My life path is controlled by forces I cannot influence”), and past-positive (e.g., “It gives me pleasure to think about my past”). A total of 25 items were measured on a 5-point Likert scale from 1 (strongly disagree) to 5 (strongly agree). We carefully examined the psychometric validity and reliability. CFA indicated that the model fits the data well: *χ*^2^(270)=5235.39, *χ*^2^/*df*=19.39, CFI=0.86, TLI=0.84, RMSEA=0.08 (90% CI: 0.077–0.080), and SRMR=0.125. The factor loadings of the items range from 0.58 to 0.78, and this measure demonstrated good internal consistency. The reliability values of the five dimensions are 0.84, 0.89, 0.80, 0.77, and 0.77, respectively.

#### Meaning in Life

Meaning in life was assessed based on the 10-item Meaning in Life Questionnaire (MLQ; Steger et al., 2006). The scale is comprised of two dimensions, namely, the presence of and the search for meaning in life. An example of the first dimension is “My life has a clear sense of purpose” and of the second dimension, “I am looking for something that makes my life feel meaningful.” All items were measured on a 7-point scale from 1 (complete non-conformance) to 5 (complete conformance). CFA indicated that the model fits the data well: *χ*^2^(33)=1,068.82, *χ*^2^/*df*=32.39, CFI=0.94, TLI=0.92, RMSEA=0.10 (90% CI: 0.097–0.108), and SRMR=0.059. In this study, this measure demonstrated good internal consistency (*α*=0.90), and the consistency coefficients of the two dimensions, the presence of meaning and the search for meaning, are 0.76 and 0.90, respectively.

### Covariates

#### Exposure Risk Level

The severity of the COVID-19 epidemic in this study was evaluated by the cumulative number of confirmed cases. All epidemic data were acquired from the official website of the National Health Commission of China.

#### Positive Affect

Positive affect was assessed based on one dimension of Positive and Negative Affect Schedule (PANAS) which is composed of 20 adjectives (Watson et al., 1988), 10 of which describe positive emotions and the other 10 describe negative emotions. Participants are asked to judge their degree of conformity with 10 positive emotional words based on their emotional state and score from 1 to 5 (1=very slightly or not at all, 5=extremely). In this study, Cronbach’s *α* coefficients was 0.90.

#### Perceived Threat

The Perceived Threat Scale was designed based on the model of risk perception by Slovic (1987) to represent perceived vulnerability and perceived severity during the outbreak of COVID-19. The six items (e.g., “I think I am very close to the epidemic in Wuhan”) were measured with a 5-point scale from 1 (strongly disagree) to 5 (strongly agree). We averaged the ratings to obtain the overall perceived threat score. Higher scores indicated a higher perceived threat. In this study, CFA indicated that the model fits the data well: *χ*^2^(9)=300.82, *χ*^2^/*df*=33.42, CFI=0.95, TLI=0.92, RMSEA=0.10 (90% CI: 0.094–0.114), and SRMR=0.036. Cronbach’s *α* coefficients was 0.82.

#### Coping Efficacy

The Coping Efficacy Scale was adapted from the Perceived Coping Efficacy Questionnaire (Kim et al., 2016). This scale was intended to measure the individuals’ belief that they and their groups could protect themselves effectively from the threat of COVID-19 Coping efficacy has two dimensions: self-efficacy and response efficacy. Four items were measured on a 5-point scale from 1 (strongly disagree) to 5 (strongly agree). We averaged the ratings to obtain the overall coping efficacy score. Higher scores indicated higher coping efficacy. In this study, Cronbach’s *α* coefficients was 0.91. CFA indicated that the model fits the data well: *χ*^2^(2)=13.05, *χ*^2^/*df*=6.53, CFI=0.99, TLI=0.99, RMSEA=0.04 (90% CI: 0.023–0.067), and SRMR=0.005.

The other covariates included in the current study were gender, income, age, educational level, and occupation.

### Analytic Strategy

We conducted mediation analysis using path analysis in Mplus 7 (Muthén and Muthén, 2000). All the models were controlled for covariates, including sex, age, income, occupation, education, exposure risk level, perceived threat, coping efficacy, and positive emotion. To assess the model fit of path analysis, we examined the chi-square test, the CFI, the RMSEA, and the SRMR (Hu and Bentler, 1999).

## Results

### Common Method Bias

As we invited one survey respondent to provide information on all the variables (i.e., maximization, time perspective, and meaning in life), this may cause common method bias. We used Harman’s single-factor test and CFA to test for the presence of common method bias. We also employed CFA to conduct the test, and the result suggested that a single-factor model did not fit the data. The CFA showed that there were seven factors with the eigenvalue higher than one and that the first factor could explain the 29.51% variance, which was lower than 40%. Thus, we determined that the common method bias in this study was not a major issue.

### Descriptive Statistics and Correlations

Means, standard deviations, reliabilities, and correlations are given in Table 1. It showed that most of correlation coefficients were moderate to strong, which indicated that there were close correlations among maximization tendency, time perspectives, and meaning in life.

**Table 1:** Descriptive statistical results of the variables.

	*M*	*SD*	1	2	3	4	5	6
1. Maximization	4.18	1.05	—					
2. TP-past-negative	3.13	0.81	0.46^***^	—				
3. TP-past-positive	3.66	0.85	0.30^***^	0.29^***^	—			
4. TP-future	3.50	0.78	0.33^***^	0.34^***^	0.63^***^	—		
5. TP-present-hedonistic	2.96	0.85	0.42^***^	0.60^***^	0.21^***^	0.19^***^	—	
6. TP-present-fatalistic	3.08	0.91	0.41^***^	0.67^***^	0.24^***^	0.24^***^	0.56^***^	
7. Meaning in Life	4.52	1.03	0.40^***^	0.24^***^	0.48^***^	0.56^***^	0.14^***^	0.13^***^

TP, time perspective. Control variables are sex, age, income, occupation, education, exposure risk level, perceived threat, coping efficacy, and positive emotion. *N*=2,987.

*** *p*<0.001.

### Multi-Mediation Model

**Figure 1:**
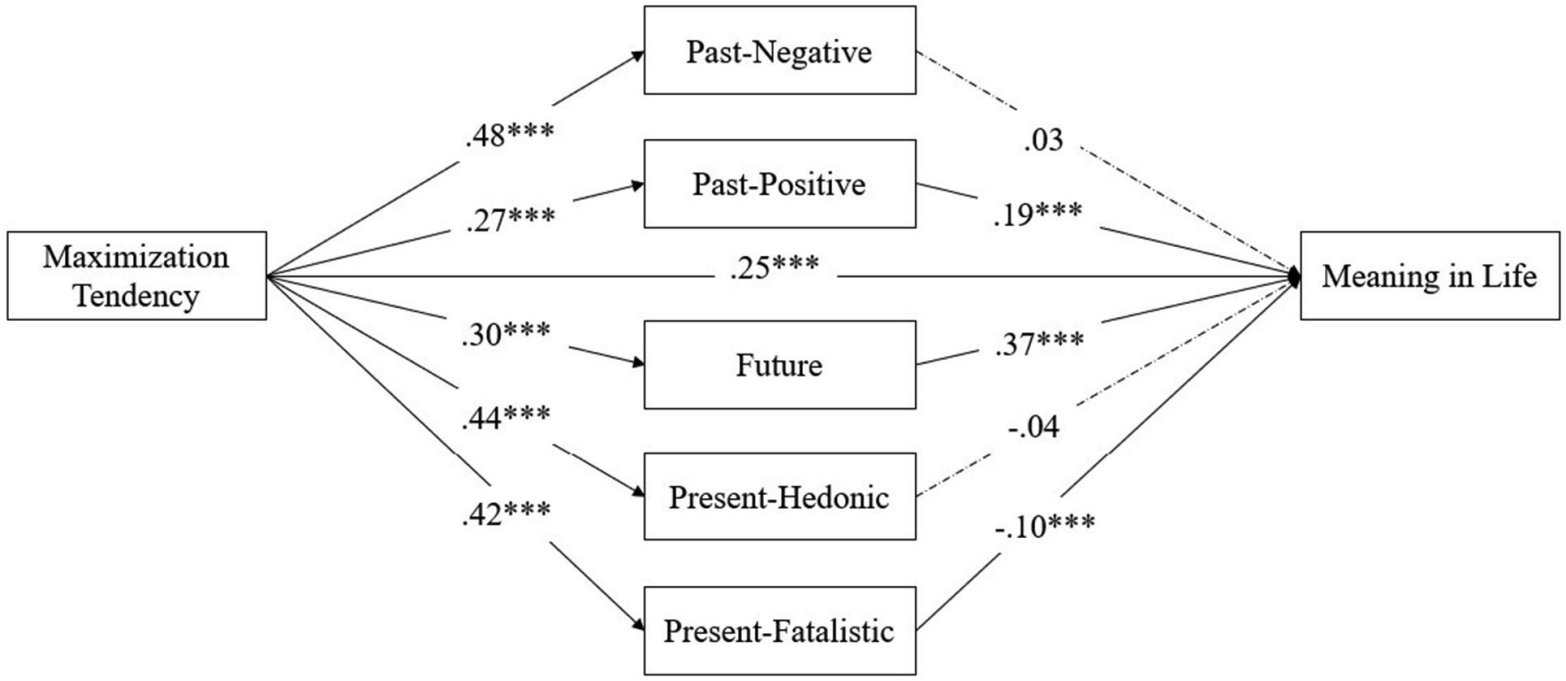
Mediating effects of time perspective between maximization tendency and meaning in life. ^***^p < 0.001.

The path coefficient estimated by the model is shown in Figure 1. We have carried out an analysis for five time perspectives. The specific mediating effect and total mediating effect are shown in Table 2. The total mediating effect value is 0.12.

**Table 2:** Mediating effects of time perspective between perfectionism and maximization.

	Estimate	*SE*	*p*	95% CI	97.5% CI
*Specific mediating effect*					
Past-negative	0.015	0.012	0.220	[−0.007, 0.034]	[−0.011, 0.037]
Past-positive	0.053	0.009	<0.001	[0.039, 0.068]	[0.036, 0.071]
Future	0.112	0.011	<0.001	[0.095, 0.130]	[0.091, 0.134]
Present-hedonistic	−0.016	0.009	0.073	[−0.031, −0.002]	[−0.033, 0.001]
Present-fatalistic	−0.043	0.010	<0.001	[−0.059, −0.027]	[−0.062, −0.024]
*Total mediating effect*					
	0.121	0.015	<0.001	[0.100, 0.148]	[0.095, 0.015]

According to the path analysis principle, five dimensions of time perspective act in mediating roles between maximization tendency and meaning in life. We explain the relationship between maximization tendency and meaning in life through five pathways (as shown in Table 2). The total effect of maximization on meaning in life is equal to the direct effect plus five indirect effects. The direct effect is 0.25, and the total indirect effect is equal to the sum of five specific mediating effects, that is, 0.12. As the mediating effects through past-negative and present-hedonistic is not significant and the pathway of present-fatalistic suppresses the relationship between maximizing and meaning in life, past-positive and future are two mediators that enhance the relationship between the two.

## Discussion

The present study examined whether and how the maximization tendency influenced the positive emotional outcome, that is, meaning in life. The results showed that the maximization tendency was positively associated with meaning in life. Specifically, the stronger the maximization tendency, the higher the level of meaning in life. On top of that, this study is the first to reveal the mechanism by which the maximization tendency is related to meaning in life. Specifically, past-positive and future time perspectives partially mediated the positive relationship between them, while a present-fatalistic perspective suppressed the positive relationship between them.

Our finding confirms the beneficial emotional impact of adopting a maximizing attitude. This finding accords with a study in the collectivist country of the Philippines (Datu, 2016). The study examines maximization as a disposition construct that reinforces the beneficial impact of positive emotion on meaning in life. The finding showed that there was a moderate correlation between maximization and meaning in life, and maximization positively moderated the link between positive affect and meaning in life. Furthermore, pertaining to the positive outcomes of maximization tendency, this finding is consistent with a few previous studies (Misuraca et al., 2016; Zhu et al., 2017), which reported that maximization was positively related to an individual’s positive outcomes, especially in numerical skills, financial plans, and outcomes.

Additionally, this relationship can be understood from the cultural perspective. In collectivist countries, tolerance of contradiction is highly encouraged (Nisbett et al., 2001) and the public are more potentially likely to withstand the cost and risk during the decision-making process (Datu, 2016). Therefore, the public are more likely to pursue the optimal choice with tremendous effort. Moreover, in a collectivist culture, the public has a greater inclination to endorse an integrative perspective of the world (Kitayama et al., 2003), which enhances the feasibility of the meaning-making process.

On the basis of our finding, the underlying mechanism is that past-positive and future time perspectives mediate the positive relationship between maximization tendency and meaning in life. In other words, they are the two mediators that account for the positive relationship between them. Theoretically, as mentioned above, construal level theory and the broaden-and-build theory of positive emotion have jointly emphasized the essential and potential roles of past-positive and future time perspectives. Based on construal level theory (Trope and Liberman, 2010), higher standards are evaluated more distantly regarding time. That is, maximizers hold higher standards, they tend to construe time more distantly and are, therefore, more likely to hold the time perspectives of past and future. The construal level theory emphasizes that psychological distance stimulates people to process events abstractly, specifically when they comprehend events in terms of the purpose rather than the actions (Vallacher and Wegner, 1987). Construing events in terms of their purpose might inspire individuals to build a greater sense of purpose in their own lives (Waytz et al., 2015). Further, in the framework of the broaden-and-build theory of positive emotion (Fredrickson, 2013), past-positive and future time perspectives will exert a positive impact on the meaning-making process since positive emotion tends to be aroused from past-positive and future-oriented time perspectives rather than past-negative.

Admittedly, there are other possible mechanisms to explain the positive mechanism between maximization tendency and meaning in life. First, on the basis of cognitive dissonance theory (Festinger, 1957), individuals adopt the cognitive strategy of rationalization to restore consonance. Many empirical studies have indicated that cognitive dissonance is a protective factor for psychological distress. Specifically, although maximizers tend to put a great deal of time and effort into identifying the optimal choice, they tend to experience more negative emotions and consequences (Schwartz et al., 2002; Iyengar et al., 2006; Newman et al., 2018). To handle dissonant experiences adequately, they are intrinsically motivated to generate or build a sense of meaning to recover the consistency. In short, it is a compensation strategy to achieve the cognitive balance. Second, the maximization tendency might bring people closer to one’s authentic self-concept, which is a potent source of meaning in life (Ryff and Singer, 2008; Waterman et al., 2008). A series of studies reveal that people who had greater cognitive accessibility of true self-concept vs. their actual selves reported greater meaning in life (Schlegel et al., 2009).

Furthermore, a present-fatalistic perspective mediated the negative relationship between maximization and meaning in life. Our results showed that present-fatalistic perspective is significantly negatively correlated with meaning in life, which is consisted with the study conducted by Chen et al. (2016). Fatalistic time perspective (FTP) represents the negative and passive temporal attributes and widely seen as being maladaptive (Zimbardo and Boyd, 1999; Worrell et al., 2016; Du et al., 2020). Studies in western countries indicated that FTP has a close correlation with low levels of SWB (Boniwell et al., 2010; Sailer et al., 2014). In eastern cultures, fatalistic propensity has been a primary element in their life (Bi and Oyserman, 2015). However, one study found out that FTP was negatively correlated with life satisfaction in China as well (Chen et al., 2016). The reason might be that they are inclined to attribute failures to fate and uncontrollable factors, which might arise a sense of helplessness that led to negative evaluation of one’s life circumstances (Du et al., 2020). Therefore, it is reasonable to understand that in Chinese culture, although fatalism propensity is a primary element in life, it is also negatively correlated with well-being. Thus, a present-fatalistic perspective plays a suppression role between maximization tendency and meaning in life.

In other words, this finding shed light on maximization paradox (i.e., the stronger the maximization tendency, the lower the level of meaning in life). Nevertheless, considering the positive relationship between maximization and meaning in life, a present-fatalistic time perspective plays the role of suppressor in the positive relationship. Thus, it is important to note that the three mechanisms work simultaneously, but the valence of the indirect effects is reversed. In summary, past-positive and future time perspectives rather than a present-fatalistic time perspective could explain the positive association between maximization and meaning in life.

Overall, this study confirmed both the beneficial aspect of maximization and the maximization paradox are possible and demonstrated the mediating role of time perspective between maximization and meaning in life. Our findings suggest that maximizing can be a good thing to enhance meaning in life. Pertaining to an individual with maximization tendency, an important means of elevating meaning in life is having a positive orientation toward your past, setting goals for future, and reducing the attitude of present-fatalism.

This study is limited in a few ways. The first limitation was the cross-cultural generalizability of research results. As we conducted the current study in the collectivist country, it remains unclear whether our results would be different in other countries. Maximization studies have indicated inconsistent results between individualist countries and collectivist countries. For example, most of the studies from individualist countries reveal that the maximization tendency is negatively correlated with life satisfaction, while there is no correlation between them in collectivist countries (Roets et al., 2012). The second limitation was that there are some criticisms on the psychometric problems and shortened versions of the ZTPI (McKay et al., 2014; Temple et al., 2019; Perry et al., 2020). Future studies should pay more attention to a theoretically driven approach to enhance the psychometric assessment and further examine the robustness of these findings. The third potential limitation was that we collected data through self-report measure. Although self-report measure has an advantage of a quick and inexpensive way to gather information, it is not always the best predictor of actual behavior (Connors et al., 2016). Future studies could apply other methodologies including experimental, neuroscience, and observational paradigms to get a deeper understanding of decision-making style and individual differences (Connors et al., 2016). For instance, movement pattern analysis (MPA) is as conceptual-based observational methodology for decoding how people differ as decision-makers with regard to cognitive motivational aspects (Connors et al., 2017; Connors and Rende, 2018) and it is an universal approach that could reliably capture and measure an individual’s style relating to the time perspective and decision-making (Connors et al., 2013, 2014). Future studies could apply MPA to decode maximizers’ movement pattern to capture their motivational and cognitional processes.

This study suggests that maximizing can be a beneficial decision style due to its positive relationships with past-positive and future-oriented thinking, which enhance individuals’ meaning in life. Thus, it is encouraged to endorse a maximizing attitude in a collectivist culture. Maximizing can be a good thing and help the individual to pursue and achieve a rational and meaningful life.

## Data Availability Statement

The raw data supporting the conclusions of this article will be made available by the authors, without undue reservation.

## Ethics Statement

The studies involving human participants were reviewed and approved by the Administration Committee of Psychological Research at the Central University of Finance and Economics. The patients/participants provided their written informed consent to participate in this study.

## Author Contributions

LZ and MM conceived and designed the study. MM performed the survey and wrote the paper. MM and NZ analyzed the data. All authors contributed to the article and approved the submitted version.

## Funding

This study was supported by the National Natural Science Foundation of China (31871120) and Program for Innovation Research in Central University of Finance and Economics.

## Conflict of Interest

The authors declare that the research was conducted in the absence of any commercial or financial relationships that could be construed as a potential conflict of interest.

## Publisher’s Note

All claims expressed in this article are solely those of the authors and do not necessarily represent those of their affiliated organizations, or those of the publisher, the editors and the reviewers. Any product that may be evaluated in this article, or claim that may be made by its manufacturer, is not guaranteed or endorsed by the publisher.

## Footnotes

^1^ https://www.wjx.cn
